# Brain endogenous angiotensin II receptor type 2 (AT2-R) protects against DOCA/salt-induced hypertension in female rats

**DOI:** 10.1186/s12974-015-0261-4

**Published:** 2015-03-08

**Authors:** Shu-Yan Dai, Wei Peng, Yu-Ping Zhang, Jian-Dong Li, Ying Shen, Xiao-Fei Sun

**Affiliations:** Department of Obstetrics and Gynecology, Shengjing Hospital, China Medical University, 36, Sanhao Street, Shenyang, 110004 China; Department of Physiology and Pathophysiology, Life Science Research Center, Hebei North University, Zhangjiakou City, Hebei China

**Keywords:** Blood pressure, Central nervous system, AT2-R, RAAS, Proinflammatory cytokines

## Abstract

**Background:**

Recent studies demonstrate that there are sex differences in the expression of angiotensin receptor type 2 (AT2-R) in the kidney and that AT2-R plays an enhanced role in regulating blood pressure (BP) in females. Also, brain AT2-R activation has been reported to negatively modulate BP and sympathetic outflow. The present study investigated whether the central blockade of endogenous AT2-R augments deoxycorticosterone acetate (DOCA)/salt-induced hypertension in both male and female rats.

**Methods:**

All rats were subcutaneously infused with DOCA combined with 1% NaCl solution as the sole drinking fluid. BP and heart rate (HR) were recorded by telemetric transmitters. To determine the effect of central AT2-R on DOCA/salt-induced hypertension, male and female rats were intracerebroventricularly (icv) infused with AT2-R antagonist, PD123,319, during DOCA/salt treatment. Subsequently, the paraventricular nucleus (PVN) of the hypothalamus, a key cardiovascular regulatory region of the brain, was analyzed by quantitative real-time PCR and Western blot.

**Results:**

DOCA/salt treatment elicited a greater increase in BP in male rats than that in females. Icv infusions of the AT2-R antagonist significantly augmented DOCA/salt pressor effects in females. However, this same treatment had no enhanced effect on DOCA/salt-induced increase in the BP in males. Real-time PCR and Western blot analysis of the female brain revealed that DOCA/salt treatment enhanced the mRNA and protein expression for both antihypertensive components including AT2-R, angiotensin-converting enzyme (ACE)-2, and interleukin (IL)-10 and hypertensive components including angiotensin receptor type 1 (AT1-R), ACE-1, tumor necrosis factor (TNF)-α, and IL-1β, but decreased mRNA expression of renin in the PVN. The central blockade of AT2-R reversed the changes in mRNA and protein expressions of ACE-2, IL-10, and renin, further increased the expressions of TNF-α and IL-1β, and kept higher the expressions of AT1-R, ACE-1, and AT2-R.

**Conclusions:**

These results indicate that endogenous AT2-R activation in the brain plays an important protective role in the development of DOCA/salt-induced hypertension in females, but not in males. The protective effect of AT2-R in females involves regulating the expression of brain renin-angiotensin system components and proinflammatory cytokines.

## Background

It is well established that there are sex differences in the development and progression of hypertension in humans and experimental animal models [1-3]. In comparison to male rodents, females are protected against angiotensin II (ANG II)- and aldosterone (Aldo)-induced hypertension [4,5]. However, the mechanism underlying this protective effect remains unclear. One of the suggested mechanisms underlying this protective effect is differences in the expression and function of the renin-angiotensin-aldosterone system (RAAS) between male and female [6]. ANG II, the main effector peptide of the RAAS exerts its effects through two main receptors, angiotensin receptor type 1 (AT1-R) and angiotensin receptor type 2 (AT2-R). AT1-R mediates the majority of physiological and pathophysiological effects of ANG II, but ANG II binding to the AT2-R appears to counteract many AT1-R-mediated effects [7]. Recent studies showed that estrogen reduces the expression of the AT1-R in target tissue but has the opposite effect on the AT2-R expression [8-11]. Ovariectomy decreases and estrogen replacement increases AT2-R expression in rat kidneys. Denton and colleagues have demonstrated that chronic infusion of a low dose of ANG II causes an increase in blood pressure (BP) in male rats, but decreases BP in female rats [12]. This depressor effect of ANG II in females is via an AT2-R-mediated and an estrogen-dependent mechanism [12]. Moreover, the chronic pressor response to ANG II was attenuated in female wild-type mice compared with male wild-type and female AT2-R-knockout mice [13]. These findings suggest that the BP is differentially regulated by the AT2-R in females as compared with males and support an enhanced role for AT2-R in regulating BP in females.

Recent studies have demonstrated that the AT2-R is expressed in several brain regions associated with cardiovascular and autonomic regulation, including the paraventricular nucleus (PVN), rostral ventrolateral medulla (RVLM), and subfornical organ (SFO) [14]. The pharmacological blockade of central AT2-R in normal animals attenuates baroreflex control of renal sympathetic nerve activity (RSNA) and heart rate (HR), indicating that endogenous AT2-R activity contributes to the normal baroreflex regulation of RSNA and HR [15]. In contrast, central AT2-R receptor activation or AT2-R overexpression in heart failure animals produces sympatho-inhibition [16,17], which was accompanied with upregulation of nNOS and downregulation of AT1-R in the several nuclei involved in the regulation of BP and sympathetic activity including the PVN [17]. These results implicate that central AT2-R plays an important role in the regulation of BP and sympathetic activity in both physiological and pathophysiological states.

Primary aldosteronism secondary to excessive and/or autonomous Aldo secretion from the RAAS accounts for approximately 10% of cases of hypertension [18]. Aldo/deoxycorticosterone acetate (DOCA)-salt hypertension is a well used animal model to mimic this human essential hypertension. Given that there is sex difference in the expression and function of AT2-R in the periphery and that AT2-R in the central nerve system regulates BP and sympathetic activity, we hypothesized that the central AT2-R may also play a protective effect against the development of DOCA/salt-induced hypertension and that its effect may be greater in females. To test this hypothesis, we employed *in vivo* telemetric recording of BP, real-time PCR, and Western blot to assess mRNA and protein expression of several RAAS components and proinflammatory cytokines in the PVN to determine the effects of the central blockade of AT2-R on the development of DOCA/salt-induced hypertension in male and female rats.

## Methods

### Animals

**Figure 1:**
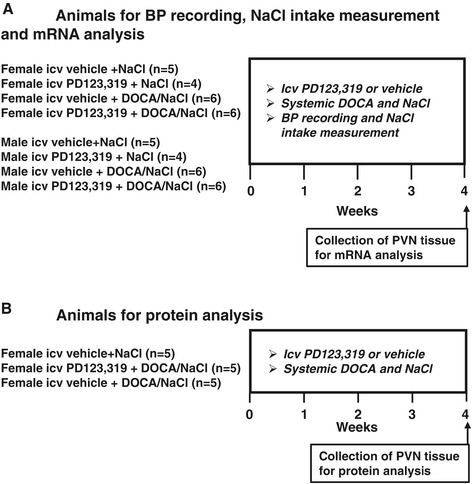
**Schematic diagram showing the animal assignment and timing of treatment. (A)** Animals for BP recording, NaCl intake measurement, and mRNA analysis. **(B)** Animals for protein analysis. Icv, intracerebroventricular; DOCA, deoxycorticosterone acetate; BP, blood pressure; PVN, paraventricular nucleus.

Thirty-six female rats and 21 male rats (Wistar, 10 to 12 weeks old) were purchased from Beijing Laboratory Animal Research Center (Beijing, China) and were maintained at an animal facility under barrier-sustained conditions with a 12-h light/dark cycle at standard conditions (temperature: 23°C ± 2°C, relative humidity: 40% to 80%) and with free access to standard rat chow and *ad libitum*. The rats were divided into eight groups and treated with an intracerebroventricular (icv) vehicle or PD123,319 (3 μg/h, GenScript), an AT2-R antagonist. The animal assignment and timing of treatment were presented in Figure 1A.

Animals assigned to DOCA treatment were subcutaneously implanted with a DOCA pellet (150 mg/kg, Sigma-Aldrich, USA). DOCA pellets were made by mixing 30- to 40-mg DOCA (adjusted by animal body weight) into 1 ml of silicone (Sylgard 184 silicone elastomer base; Dow Corning, Midland, MI). Once the DOCA was homogenously mixed into the silicone, a silicone elastomer curing agent (0.2 ml) was added. The DOCA implants were allowed to cure at room temperature for 24 h and were then stored at 4°C until implantation. Water was also changed to 1% NaCl as the sole drinking fluid. The 1% NaCl intakes were measured daily. After the physiological studies were finished, brains were taken and PVN tissue was collected by micropunching for determining mRNA expression of several components of RAAS and proinflammatory cytokines. The brains from three additional groups given the same treatments as those in intact females without blood pressure recording were also taken for determining protein expression (Figure 1B, *n* = 5 each group).

All animal procedures were reviewed and approved by the China Medical University and the Hebei North University Institutional Animal Care and Use Committee conforming to US National Institutes of Health guidelines.

### Telemetry probe implantation

Under anesthetization with pentobarbital sodium (1%, 50 mg/kg), rats were implanted with telemetry transmitters (TA11-PA40, DSI) through the femoral artery for continuous monitoring of mean arterial pressure (MAP) and HR.

### Chronic icv cannula and osmotic pump implantation

After baseline, BP and HR recordings were made, the rats were again anesthetized with pentobarbital sodium, and the icv cannula with an osmotic pump (ALZET Brain Infusion Kits, Alzet Co.) was implanted into the right lateral ventricle (the coordinates 1.0 mm caudal, 1.5 mm lateral to the bregma, and 4.5 mm below the skull surface) for chronic infusion of vehicle or PD123,319 for 4 weeks. At the same time, a pellet of DOCA (150 mg/kg) was implanted subcutaneously in the back and tap water was changed to 1% NaCl.

### Micropunch of the PVN

At the end of the experiments, the animals were euthanized with an overdose of pentobarbital. The brain was removed and quickly frozen on dry ice. Six serial coronal sections (100 μm) were cut through the hypothalamus at the level of the PVN using a cryostat, and the PVN region was punched using a blunt 18-gauge needle as previously described [19].

### Real-time PCR analysis

The total RNA was extracted using RNeasy® Mini Kit (Qiagen, Valencia, CA, USA) and reverse transcribed into cDNA. mRNA levels for renin-angiotensin system components (renin, AT1-R, AT2-R, angiotensin-converting enzyme (ACE)-1, and ACE-2) and inflammatory cytokines (tumor necrosis factor (TNF)-α, interleukin (IL)-1β, and IL-10) and glyceraldehyde 3-phosphate dehydrogenase (GAPDH) were analyzed with SYBR Green real-time PCR. The sequences for the primers are summarized in Table 1. Real-time PCR was performed with the ABI prism 7300 Sequence Detection System (Applied Biosystems, Carlsbad, CA). The values were corrected by GAPDH, and the final concentration of mRNA was calculated using the formula *x* = 2^−ΔΔCt^, where *x* = fold difference relative to control.

**Table 1:** **Sequences for primers**

**Gene**	**Gene ID**	**Primers**	**Sequences**
Renin	NM_012642	Forward	5′-CAGGAACGATGACCTGTGCAT-3′
		Reverse	5′- CAGTGGGTGGTGGGATGTC-3′
AT1-R	NM_030985	Forward	5′-CTCAAGCCTGTCTACGAAAATGAG -3′
		Reverse	5′-GTGAATGGTCCTTTGGTCGT -3′
AT2-R	NM_012494	Forward	5′-TGCTGTTGTGTTGGCATTCA-3′
		Reverse	5′-ATCCAAGAAGGTCAGAACATGGA-3′
ACE-1	NM_012544	Forward	5′-TTTGCTACACAAATGGCACTTGT-3′
		Reverse	5′-CGGGACGTGGCCATTATATT-3′
ACE-2	NM-001012006	Forward	5′-TTGAACCAGGATTGGACGAAA-3′
		Reverse	5′-GCCCAGAGCCTACGATTGTAGT-3′
TNF-α	NM_013693	Forward	5′-GCATGATCCGCGACGTGGAA-3′
		Reverse	5′-AGATCCATGCCGTTGGCCAG-3′
IL-1β	NM_031512	Forward	5′-TGA TGT TCC CAT TAG ACA GC-3′
		Reverse	5′-GAG GTG CTG ATG TAC CAG TT-3′
IL-10	NM_012854	Forward	5′-GTTGCCAAGCCTTGTCAGAAA-3′
		Reverse	5′-TTTCTGGGCCATGGTTCTCT-3′
GAPDH	NM_017008	Forward	5′-GCCAAAAGGGTCATCATCTC-3′
		Reverse	5′-GGCCATCCACAGTCTTCT-3′

AT, angiotensin receptor type; ACE, angiotensin-converting enzyme; IL, interleukin; TNF, tumor necrosis factor; GAPDH, glyceraldehyde 3-phosphate dehydrogenase.

### Western blot analysis

The PVN tissue was homogenized in a lysis buffer, and the protein concentration in the supernatant was measured with the BCA protein assay Kit (Pierce, Rockford, IL, USA). Equivalent amounts of protein were separated on 12% SDS-polyacrylamide gels and transferred to polyvinylidene difluoride membranes (Millipore Corporation, Bedford, MA, USA). The membranes were blocked with 5% nonfat dry milk and then incubated using a primary antibody at 4°C overnight. The following primary antibodies used in this study have been verified in previous published literatures: anti-renin (sc-22752) [20], anti-AT1-R (sc-1173) [21,22], anti-AT2-R (sc-9040) [23], anti-ACE-1 (sc-20791) [22], anti-ACE-2 (sc-20998) [24], anti-TNF-α (sc-1350) [25], anti-IL-1β (sc-7884) [25], anti-IL-10 (sc-57245) [26], and anti- β-actin (sc-47778) [22] (all antibodies were purchased from Santa Cruz Biotechnology Inc., Santa Cruz, CA). After three washings, the membranes were incubated with horseradish peroxidase-conjugated second antibody (Santa Cruz Biotechnology Inc, Santa Cruz, CA) for 1 h at room temperature. The signal was visualized using the enhanced chemiluminescence (ECL) detection system (Amersham), and the densities of the immunobands were quantitated using NIH ImageJ software (Bethesda, MD, USA). All data were corrected by β-actin.

### Data analysis

MAP and HR are presented as mean daily values. Differences for MAP and HR were calculated for each animal based on the mean of the 4-day baseline subtracted from the mean of the final 5 days of treatment. Two-way ANOVA analysis for the experimental groups was then conducted on the means of the calculated differences (the factors were sex (female and male) and treatment (systemic infusion of DOCA with or without icv AT2-R antagonist)). After establishing a significant ANOVA, *post hoc* analyses were performed with Tukey multiple comparison tests between pairs of mean changes. The same statistical methods were used to analyze the changes in HR, 1% NaCl intake, and differences in mRNA and protein expression of the RAAS components and cytokines in the PVN. All data are expressed as means ± SE. Statistical significance was set at *P* < 0.05.

## Results

### Effect of icv infusion of AT2-R antagonist PD123,319 on DOCA/salt-induced hypertension in female rats

**Figure 2:**
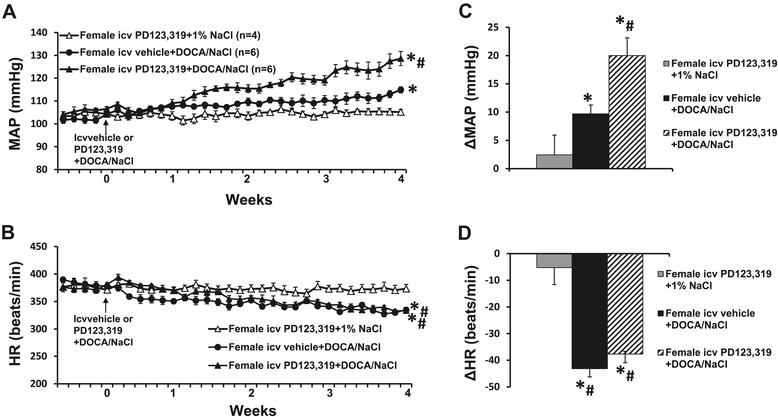
**The effect of central infusion of AT2-R antagonist PD123,319 on DOCA/salt-induced hypertension in female rats.** Daily mean arterial pressures (MAP) **(A)** and heart rate (HR) **(B)** before and during DOCA/salt treatments in intact females with or without central infusions of PD123,319. **(C,D)**. Average changes in MAP and HR induced by DOCA/NaCl treatment in all groups. *P < 0.05 compared to baseline, ^#^ *P* < 0.05 compared to females with central infusion of PD123,319. DOCA, deoxycorticosterone acetate; icv, intracerebroventricular.

The 1% NaCl alone (data not shown in Figure 2) or icv infusion of PD123,319 plus 1% NaCl had no effects on the basal MAP (104.6 ± 1.5 mmHg) and HR (375.9 ± 7.8 beats/min) in female rats. DOCA/salt treatment elicited a slight, but significant, increase in MAP in females with icv vehicle infusion (∆9.7 ± 1.8 mmHg, *P* < 0.05). Icv infusions of PD123,319 significantly augmented these DOCA/salt pressor effects (∆20.0 ± 3.2 mmHg, *P* < 0.05, Figure 2A,C). In contrast, systemic DOCA infusion produced a significant, comparable decrease in HR (Figure 2B,D, *P* > 0.05) in all groups when compared to rats given PD123,319 plus 1% NaCl.

### Effect of icv infusion of PD123,319 on DOCA/salt-induced hypertension in male rats

**Figure 3:**
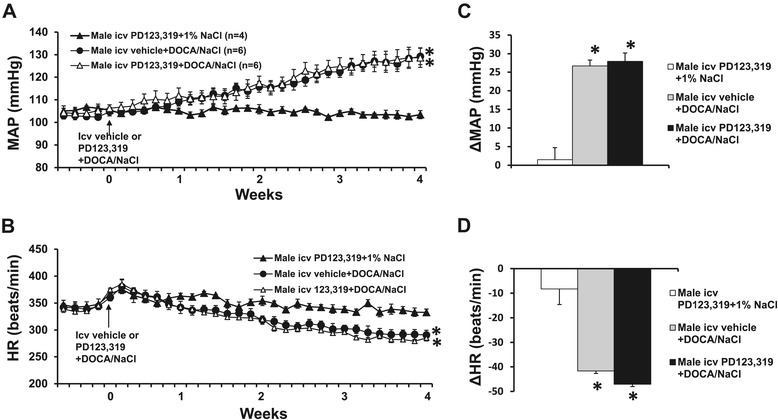
**The effect of central infusion of AT2-R antagonist PD123,319 on DOCA/salt-induced hypertension in male rats.** Daily mean arterial pressures (MAP) **(A)** and heart rate (HR) **(B)** before and during DOCA/salt treatments in male rats with or without central infusions of PD123,319. Average changes in MAP and HR induced by DOCA/salt treatment in all groups **(C,D)**. *P < 0.05 compared to females with central infusion of PD123,319 alone. DOCA, deoxycorticosterone acetate; icv, intracerebroventricular.

The 1% NaCl alone (data not shown in Figure 3) or icv infusion of PD123,319 plus 1% NaCl had no effects on the basal MAP (105.1 ± 1.8 mmHg) and HR (349.4 ± 7.5 beats/min) in males. After 28 days of DOCA/salt treatment, the MAP was significantly increased in males (∆25.7 ± 2.3 mmHg, *P* < 0.05 vs baseline and female group with icv vehicle plus systemic DOCA). However, icv infusion of PD123,319 for 4 weeks did not change the DOCA/salt pressor effect (∆26.1 ± 3.8 mmHg, *P* < 0.05, Figure 3A,C). Moreover, the increases in blood pressure in these male groups were not significantly different from that in the female group with the central PD123,319 plus systemic DOCA (*P* > 0.05). Systemic DOCA infusions also produced a significant, comparable decrease in HR in all groups (Figure 3B,D).

### Effects of DOCA infusion on 1% NaCl intake in males and females with icv infusions of PD123,319

**Figure 4:**
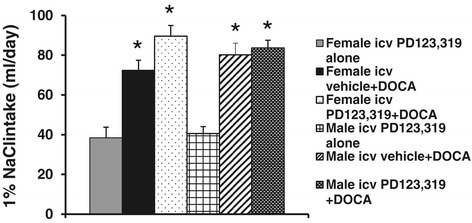
**Mean daily 1% NaCl intake during DOCA infusions in male and female rats treated with central vehicle or PD123,319.** *P < 0.05 compared to females or males with central infusion of PD123,319 alone. DOCA, deoxycorticosterone acetate; icv, intracerebroventricular.

There was no difference in the 1% NaCl intake between male and female rats when given icv infusions of PD123,319 alone. Systemic infusion of DOCA produced a significant, but comparable, increase in 1% NaCl intake in all groups of rats (Figure 4).

### The effect of DOCA infusion on the mRNA expression of RAAS components and inflammatory cytokines in the PVN of female rats

**Figure 5:**
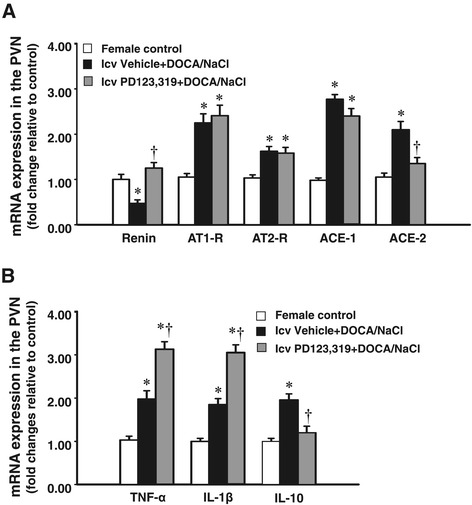
**mRNA levels for renin-angiotensin system components (renin, AT1-R, AT2-R, ACE-1, ACE-2) (A) and inflammatory cytokines (TNF-α, IL-1β, and IL-10) (B) in the PVN in each group of intact females.** Values are mean ± SEM (*n* = 4 to 6 for each group) and expressed as a fold change relative to corresponding control. *P < 0.05 compared to control females, ^†^ *P* < 0.05 compared to central vehicle plus systemic DOCA/salt. DOCA, deoxycorticosterone acetate; icv, intracerebroventricular; PVN, paraventricular nucleus; AT, angiotensin receptor type; ACE, angiotensin-converting enzyme; IL, interleukin; TNF, tumor necrosis factor.

In the basal state, female rats possessed a slight, but not significant, increase in mRNA expression of AT2-R in the PVN (1.25 ± 0.09 fold, *P* > 0.05) when compared to males. However, the DOCA/salt treatment induced a significant increase in the mRNA expression of PVN AT2-R only in female rats (*P* < 0.05, Figure 5A), but not in male rats (data not shown).

In the PVN of the female, DOCA also induced a significant increase in the mRNA expression of AT1-R, ACE1, and ACE2, but a significant decrease in the mRNA expression of renin (*P* < 0.05). Central infusion of PD123,319 reversed the changes in the mRNA expression of renin and ACE2, showing that decreased renin expression was elevated while the increased ACE2 expression was reduced during DOCA infusion. The mRNA expression of AT1-R, AT2-R, and ACE1 remained higher (Figure 5A). Likewise, DOCA infusion significantly increased the mRNA expression of TNF-α, IL-1β, and IL-10. The central infusion of PD123,319 reversed the change in the mRNA expression of IL-10, but further augmented the mRNA expression of TNF-α and IL-1β (Figure 5B).

### The effect of DOCA infusion on the protein expression of RAAS components and inflammatory cytokines in the PVN of female rats

**Figure 6:**
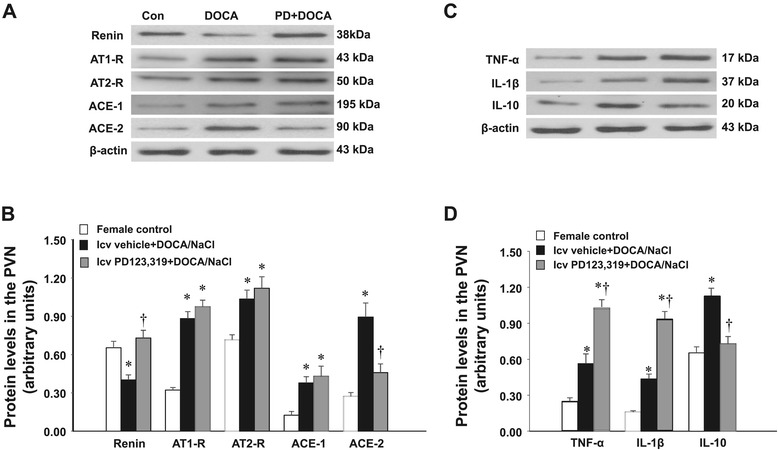
**Representative Western blots and quantitative comparison of protein levels for renin-angiotensin system components (renin, AT1-R, AT2-R, ACE-1, ACE-2) (A,B) and inflammatory cytokines (TNF-α, IL-1β and IL-10) (C,D) in the PVN in each group of intact females.** Values are corrected by β-actin and expressed as mean ± SEM (*n* = 5 for each group). *P < 0.05 compared to control females, ^†^ *P* < 0.05 compared to central vehicle plus systemic DOCA/salt. Con, control; DOCA, deoxycorticosterone acetate; icv, intracerebroventricular; PVN, paraventricular nucleus; AT, angiotensin receptor type; ACE, angiotensin-converting enzyme; IL, interleukin; TNF, tumor necrosis factor.

The Western blotting analysis for protein expression confirmed the effects of DOCA and AT2-R antagonist on the genomic regulation in females. DOCA treatment resulted in a significant increase in the protein expression of AT1-R, AT2-R, ACE1, ACE2, TNF-α, IL-1β, and IL-10 in the PVN while renin was decreased (*P* < 0.05). The central infusion of PD123,319 reversed the changes in the protein expression of renin, ACE2, and IL-10, augmented the protein expression of TNF-α and IL-1β, and kept higher the protein expression of AT1-R, AT2-R, and ACE1 during DOCA infusion (*P* < 0.05, Figure 6).

## Discussion

The major findings of the present study are that 1) the central blockade of AT2-R augmented the DOCA/salt-induced pressor effect in females, but not in males; 2) in DOCA/salt-treated female rats, increased mRNA and protein expression of AT1-R, ACE1, TNF-α, and IL-1β in the PVN may contribute to increased BP, but increased expression of AT2-R, ACE2, and IL-10 in the PVN may play a protective role against the BP increase; and 3) during icv infusion of AT2-R antagonist, enhanced mRNA and protein expression of renin, TNF-α, and IL-1β, and decreased ACE2 and IL-10 expression in the PVN may be responsible for the augmentation of pressor effects induced by DOCA/salt in females. These results suggest that AT2-R in the brain plays a protective role in the development of DOCA/salt-induced hypertension in females and that this protective effect is via modulation of PVN expression of RAAS components and proinflammatory cytokines.

Within the RAAS, ACE1/ANG II/AT1-R has been considered as a hypertensive axis while ACE2/ANG-(1 to 7)/Mas-R and ANG II/AT2-R have been viewed as an antihypertensive axis [27]. It is well established that activation of the AT2-R provides a counter-regulatory role to AT1-R overactivity, particularly during pathological conditions [28]. AT2-R also interacts with ANG-(1 to 7), another important component of the antihypertensive axis, to regulate the vasorelaxation and BP [29]. Early studies showed that global AT2-R knockout mice had slightly elevated BP as compared to wild-type control mice and that ANG II or DOCA/salt induced a greater and steeper increase in BP in AT2-R knockout mice [30]. In contrast, central injection of ANG II plus the AT2-R antagonist PD123,319 initiated a greater pressor response than the one induced by ANG II alone in wild-type mice [31]. Moreover, Gao and colleagues reported that there is robust expression of AT2-R in the brain and that central overexpression or activation of AT2-R by Compound 21 (C21), the first selective nonpeptide AT2-R agonist, reduces sympathetic outflow and BP in male rats [14,17,32]. These results suggest that AT2-R has a negative influence on neuronal function and cardiovascular activity, which buffers the stimulating effect of AT1-R. However, inconsistent to these abovementioned results, we did not find the enhanced effect of the central blockade of AT2-R on BP in normotensive or DOCA/salt-treated male rats in the present study, suggesting that the central AT2-R does not tonically regulate basal BP and has no protective effect on the development of DOCA/salt-induced hypertension in male rats. Indeed, recent studies showed that AT2-R stimulation *in vivo* does not seem to have a BP-lowering effect in male spontaneously hypertensive rats. Because AT1-R plays a permanent and dominating role in controlling cardiovascular function, the BP-lowering effect of the AT2-R can be unmasked only when C21 is combined with a low-dose, almost BP-neutral AT1-R blocker [33]. Recently, Gao *et al.* reported that AT2-R protein expression was significantly downregulated in the RVLM of male rats with chronic heart failure and that this decrease in AT2-R expression played a critical role in sympathoexcitation in this syndrome [34]. Thus, it is possible that AT2-R was also downregulated during the DOCA/salt treatment and that the depressor effect of AT2-R was reduced and covered by the dominating pressor effect of AT1-R in male rats in the present study. Moreover, the discrepancies between the present study and the aforementioned results may also be attributed to the differences in manipulating sites of AT2-R or in pharmacological applications (global AT2-R knockout or exogenous activation of central AT2-R by potent agonist vs only the blockade of central endogenous AT2-R in the present study).

Chronic ANG II- or Aldo-induced hypertension is attenuated in females as compared to male rodents [4,5]. Denton and colleagues have demonstrated that females have a higher expression of AT2-R in the kidneys, which is responsible for lower BP response to ANG II as compared to males [12]. AT2-R deficiency increases the ANG II pressor effect in female mice to a similar extent to that observed in male mice [13]. These results indicate that AT2-R plays a sex-specific role in regulating BP, with an enhanced effect in females. In the present study, we found that the central blockade of AT2-R augmented the DOCA/salt-induced pressor effect in females, suggesting that endogenous activation of the central AT2-R during DOCA/salt treatment plays a protective effect in the development of hypertension. Rodriguez-Perez and colleagues reported that the basal mRNA and protein expressions of AT2-R in the substantia nigra were higher in females with a high level of estrogen during the estrous cycle than in males [35]. Estrogen replacement reversed the ovariectomy-induced decrease in AT2-R expression in the same nucleus [10]. However, in the present study, the basal expression of AT2-R only exhibited a slight, but not significant, increase in females when compared to males. The discrepancy for this basal expression of AT2-R in females may be due to the difference in nucleus studied or the different period of estrous cycle when killed (only 12 h with a high level of estrogen in a 4-day estrous cycle in rat). Nevertheless, we found that the mRNA and protein expression of AT2-R was upregulated in the PVN during the DOCA/salt infusion in females, which implies that the increased expression of AT2-R plays a role in antagonizing the pressor effect produced by the DOCA/salt treatment in females. The present study extends the previous studies focusing on the effect of AT2-R in peripheral tissues and provides a new central mechanism responsible for the protective effect of AT2-R in females.

In addition, although male rats showed a greater increase in BP response to DOCA than female rats, and the central AT2-R only changed the BP responses to DOCA infusion in female rats in the present studies, saline intakes in all groups were similar regardless of sex or treatment condition. Thus, the sex differences in DOCA-induced hypertension and the effects of central AT2-R on DOCA-induced hypertension in the present study are unlikely to be due to saline intake, which is consistent with the previous studies [5].

The RAAS components apart from having independent effects also influence the function and generation of other RAAS components. These converging effects contribute to the net changes in cardiovascular function and BP. For example, in obese Zucker rats, long-term AT2-R activation increased kidney ACE2 expression and activity, the Mas receptor (MasR), and its ligand ANG-(1 to 7), but attenuated AT1-R expression and BP [36]. The AT2-R activation has also been reported to exert a negative feedback on renin production [37]. It has been established that there are sex differences in the expression levels of components of the RAAS, with upregulation of the antihypertensive axis including ACE2/ANG-(1 to 7)/Mas-R and AT2-R in females. In wild-type mice, AT2-R expression in the injured artery was enhanced, and this increase was greater in female than in male mice [38]. Moreover, the AT2-R overexpression downregulated AT1-R expression in female mesangial cells while AT2-R transfection upregulated AT1-R expression in male cells [24]. These results indicate that there is a different way in the RAAS that male and female respond to physiological and pathophysiological stimulations, and the female shifts the balance of the RAAS to favor the antihypertensive axis. In the present study, we found that in female rats, DOCA/salt treatment upregulated not only the expression of AT1-R and ACE1, but also expression of AT2-R and ACE2 in the PVN while the renin expression was decreased. Given the counter-regulatory effects of the AT2-R and ACE2 on AT1-R and ACE1 overactivity, the increased expression of AT2-R and ACE2 and decreased expression of renin may play a protective role in antagonizing the overactivity of AT1-R and ACE1 in the development of DOCA/salt-induced hypertension in female rats. In contrast, the central blockade of AT2-R reversed the changes in the mRNA and protein expression of renin and ACE2, and AT1-R and ACE1 were kept higher in the PVN during the DOCA/salt treatment, suggesting that the protective role of ACE2 was lost, but prohypertensive factors such as renin, AT1-R, and ACE1 were still presented, thereby contributing to the enhanced increase in blood pressure under condition of AT2-R antagonism. Taken together, these results indicate that enhanced AT2-R during DOCA/salt treatment can upregulate other components of the antihypertensive axis (ACE2), but downregulate the component of hypertensive axis (renin) to play a protective role against the BP increase induced by DOCA/salt in females.

Overactivity of the proinflammatory cytokines such as TNF-α and IL-1β in the PVN, a critical cardiovascular and autonomic center, has been implicated in the development and the maintenance of hypertension and heart failure [39,40]. Both the proinflammatory cytokines and RAAS can positively regulate each other to facilitate this process [40]. In contrast, IL-10 in the PVN plays a protective role against hypertension and heart failure [41,42]. Besides the effects of AT2-R on the RAAS, there is evidence suggesting that AT2-R exerts anti-inflammatory activity which is another contributor to the beneficial effect of AT2-R. Hussain *et al.* have recently shown that AT2-R stimulation exerts a novel anti-inflammatory response in renal epithelial cells and THP-1 macrophages via reduced TNF-α and enhanced IL-10 production. IL-10 was critical for the anti-inflammatory effects of AT2-R stimulation because the IL-10-neutralizing antibody dose-dependently abolished the AT2R-mediated decrease in TNF-α level [43,44]. In *in vivo* experiments, AT2-R activation decreased levels of TNF-α in obese Zucker rats. Conversely, the blockade of AT2-R by PD123,319 significantly lowered the renal IL-10 levels [43]. These results suggest that AT2-R exerts the beneficial effect on cardiovascular function via regulation of inflammatory cytokines.

Recent studies demonstrate that estrogen treatment significantly inhibits brain AT1-R and ACE1 expression and proinflammatory cytokine production induced by ovariectomy [10,11], severe burn injury, or trauma-hemorrhage [45,46]. Estrogen also increases IL-10 and decreases TNF-α release from resting and LPS-stimulated N9 microglial cells [47]. Denton *et al.* recently demonstrated a key role for the AT2-R in the normal regulation of BP during pregnancy. AT2-R deficiency resulted in a phenotypic switch in the T cells infiltrating the kidney toward a proinflammatory phenotype, thereby increasing the BP during late gestation [48]. These results suggest that estrogen can interact with AT2-R to improve neural and cardiovascular function through their anti-inflammatory effect. In the present study, female rats showed an enhanced expression of TNF-α and IL-1β and a decreased expression of IL-10 in the PVN when central AT2-R was blocked during DOCA/salt treatment, suggesting that the anti-inflammatory effect of central AT2-R may play an important protective role in the development of DOCA/salt-induced hypertension. Furthermore, given the reciprocal enhancement of the RAAS and the proinflammatory cytokines, the AT2-R regulation of both the RAAS and the proinflammatory cytokines may play a synergistic role in antagonizing the increase in blood pressure produced by DOCA/salt in females.

## Conclusions

The central blockade of AT2-R enhanced hypertensive components and inhibited antihypertensive components in the brain nucleus involved in regulation of cardiovascular function, thereby increasing the BP induced by DOCA/salt in females. The results indicate that central AT2-R in females plays an enhanced protective role through modulation of RAAS and inflammatory cytokines. The present findings may also have important implications that targeting AT2-R is a new approach for the prevention and treatment of human hypertension.
